# Relationship Between Occupational Exposure to Noise and Vibrations and Vertigo: A Prospective Case-Control Study

**DOI:** 10.3390/jcm13226650

**Published:** 2024-11-06

**Authors:** Inés Sánchez-Sellero, Andrés Soto-Varela

**Affiliations:** 1 Division of Toxicology, Department of Forensic Sciences, Pathology, Gynecology and Obstetrics, and Pediatrics, School of Medicine, Forensic Sciences Institute, Universidade de Santiago de Compostela, 15782 Santiago de Compostela, Spain; ines.sanchez.sellero@usc.es; 2 Division of Neurotology, Department of Otorhinolaryngology, Complexo Hospitalario Universitario de Santiago de Compostela, 15706 Santiago de Compostela, Spain; 3 Department of Surgery and Medical-Surgical Specialities, School of Medicine, Universidade de Santiago de Compostela, 15782 Santiago de Compostela, Spain; 4 Health Research Institute of Santiago (IDIS), 15706 Santiago de Compostela, Spain

**Keywords:** vertigo, occupation, noise, vibration, Meniere’s disease, BPPV

## Abstract

**Background/Objectives**: It is known that balance disorders involve occupational hazards. However, the inverse relationship (between certain occupations and an increased incidence of vertigo or dizziness) has been scarcely studied. The objective of this work was to analyze the occupation of a group of patients with vertigo compared to the economically active general population and to evaluate the prevalence of occupational noise and/or vibration exposure in both groups. **Methods**: A prospective cross-sectional, observational, case-control study was carried out, including 393 patients (193: Meniere’s disease; 63: vestibular migraine; 21: vestibular neuritis; 116: BPPV) (244 women and 149 men). These patients were compared to a control group from the general population obtained from 6th EWCS-Spain (2015). Possible differences regarding sex, age, occupation, exposure to noise, and exposure to mechanical vibrations were analyzed. **Results**: Differences in the distribution of occupations between patients with vertigo and the general population were observed (Chi-square, *p* = 4.065 × e^−20^). Patients with vertigo were significantly more exposed to noise (Fisher’s exact test, *p* = 2.97 × e^−10^; OR = 2.595, CI95% (1.916;3.515)) and vibrations (Fisher’s exact test, *p* = 6.23 × e^−10^; OR = 2.722, CI95% (1.963;3.775)) than the control group. These differences were observed both between men and women. **Conclusions**: A relationship between occupational exposure to noise and/or vibrations and the presence of vertigo was observed. Protective and preventive measures could help prevent the occurrence of some diseases involving vertigo.

## 1. Introduction

Vertigo and balance disorders are some of the most frequent reasons for consultation in Primary Care and Otorhinolaryngology. Dizziness is suggested to handicap the patients significantly, especially in daily activities. Moreover, the fluctuating occurrence of symptoms combined with an unpredictable evolution of balance disorders over time explains a higher hazard in patients with certain occupations (e.g., road hauliers, bus drivers, or workers at heights) or, at least, emotional distress related to this risk.

We hypothesized that, in addition to clinical variables (e.g., severity, duration, and frequency of vertigo attacks) and associated symptoms (e.g., nausea, vomiting, hearing loss, headache, and photophobia), environmental factors influence the quality of life in patients who experience vertigo or disequilibrium. Might these factors (such as diet, weather changes, marital status, stress and anxiety, or occupation) influence clinical features, emotional functioning, quality of life, and even the ability to carry out their work activities? As these factors are external variables, the introduction of measures to change some of them is relatively easy. However, other ones are not likely to be modified. Recent studies on the putative relationship between caffeine consumption [1], alcohol consumption [2], atmospheric pressure changes [3] or stress [4], and clinical features of Menière’s disease were published.

It is known that balance disorders involve occupational hazards, even life-threatening ones. To date, however, the inverse relationship (between certain occupations and an increased incidence of vertigo or dizziness) has been scarcely studied.

It is well known that exposure to dangerous working environments at the workplace may result in diseases—so-called “occupational diseases”. In Spain, “noise-induced hearing loss” is the only occupational disease related to the inner ear recognized. There are scarcely any studies on a putative relationship between occupation and vertigo. Consistent with this situation, no balance disorder has been recognized as an “occupational disease”. However, we believe that the putative association must be studied to improve the knowledge of balance disorders and to introduce measures to encourage improvements in the safety, health, and clinical management of patients affected. Moreover, we consider that further studies are required to evaluate the association between noise exposure and vestibular damage because this question has been little studied [5,6,7,8]. Recent studies suggest an exposure-response association between exposure to strong static magnetic fields and transient symptoms such as vertigo among healthcare and research staff [9]. In vivo, animal models confirmed that some utricular and saccular afferents can be evoked by both air-conducted sound and bone-conducted vibration [10]. This is in accordance with the physiopathological basis of vestibular evoked myogenic potentials (VEMPs); air-conducted sound or bone-conducted vibration stimulations are used in the clinic to evoke vestibular responses, which can be recorded and measured. Several cases of benign paroxysmal positional vertigo have been reported in skeet shooters and hunters [11], considering that the transmission of impulsive and vibratory energy from the shoulder may be responsible for the otoconia detachment. In an extensive review conducted on the adverse health effects associated with occupational exposure to vibration [12], the presence of dizziness and motion sickness was described, but no specific association between vibrations and vestibular diseases was described. In addition, we hypothesized that exposure to mechanically generated vibrations might also be associated with balance disorders by mechanistic origin (e.g., benign paroxysmal positional vertigo because of detachment of otoconia). There are only a few publications that study this relationship [13].

Based on the previously mentioned, the main objective of our study was to analyze the occupation of a group of patients with vertigo, compared to the economically active general population; specifically, we intend to study whether our group of patients showed occupations that require greater physical activity or exposure to potentially aggressive external factors (and that could be etiological agents of vertigo). As secondary objectives, we proposed to analyze the prevalence of occupational noise exposure and occupational vibration exposure in patients with vertigo, compare it to that observed in economically active populations, and evaluate the aforementioned data jointly, including women and men, distinguished by gender.

## 2. Materials and Methods

### 2.1. Design

This is a prospective cross-sectional, observational, case-control study. Data were prospectively recorded for three years (2021–2023). All participants were recruited from patients affected by vertigo who had been referred to the Neurotology Unit of a tertiary hospital, the Complexo Hospitalario Universitario de Santiago, in Santiago de Compostela (Spain).

### 2.2. Inclusion Criteria

Patients with a definite diagnosis of any of the following four disorders were asked to participate in this study:

- Definite Menière’s disease (MD), according to the Classification Committee of the Bárány Society [14];
- Definite Vestibular Migraine (VM), according to the Classification Committee of the Bárány Society [15];
- Vestibular Neuritis (VN) (acute vestibular syndrome, with objective data of unilateral vestibulopathy, without hearing affectation and without data of central lesions);
- Benign Paroxysmal Positional Vertigo (BPPV), according to Bárány Society criteria [16].

### 2.3. Exclusion Criteria

The following exclusion criteria were applied:

- Patients < 18 years old;
- Patients with vertigo of two or more causes simultaneously;
- Patients with systemic diseases or locomotor pathologies that had conditioned the choice of their occupations;
- Patients affected by severe cognitive decline that prevented them from obtaining informed consent.

### 2.4. Methodology

In order to establish a diagnosis, all participants with vertigo underwent a complete neuro-otologic examination, including otoscopy, basic neurologic exploration, observation (and, in some cases, record) of spontaneous nystagmus, head-impulse test, positional tests (Dix and Hallpike, McClure, and cephalic hyperextension tests), and pure tone audiometry. When they were necessary, videonystagmography (with caloric tests) and vestibular-evoked myogenic potentials (VEMPs) were performed. All patients diagnosed with Menière’s disease underwent encephalic MRI with the aim of excluding other causes of their symptoms.

Variables obtained and studied from all patients were the following:

- Age;
- Sex;
- Diagnosis.
- Occupation. Anamneses included occupational history. The participants were asked about all their jobs throughout their lives (not just their current ones). The main job (one that they had dedicated the most time of their working life to) was registered;
- Occupational exposure to noise;
- Occupational exposure to vibrations.

The occupations of patients and control subjects were classified into ten major groups according to the International Standard Classification of Occupations, 2008 (ISCO-08) and its Spanish version (National Classification of Occupations NCO-11 2010) [17]. This occupational classification system distinguishes ten major groups (Table 1).

In addition to these ten groups, another group was added to our study. This new group included “housewives, students, and unemployed people”. On the other hand, retired people were also included in this study, recording the main occupation in their working lives. In Spain, the Social Security National Institute (SSNI) published in 2014 the third edition of the “Occupational Evaluation Guide” [18], including 502 specific descriptions of all occupations recognized by NCO-11. These 502 data sheets provide information on a broad range of issues, including “risk factors and specific working conditions”. These factors and conditions were classified into three categories (possible risks from the work environment, possible risks from the use of tools or machinery, and specific circumstances of the workplace). We select the following:

- Possible risks from the work environment. Our study assessed one of them: “exposure to noise”;
- Possible risks from the use of tools or machinery. Our study selected “exposure to vibrations from tools or machinery”.

Data on exposure or no exposure to occupational noise were obtained in all participants. We assumed that a subject was exposed to occupational noise if this exposure was included as a risk to the work environment in the SSNI specifications corresponding to his occupation. For all patients, information related to possible occupational exposure to mechanically generated vibrations was also obtained from the SSNI datasheet for each occupation. In addition to recording their main occupation, the patients were specifically asked about occupational exposure to noise and/or vibrations, thus complementing the information obtained from the SSNI.

### 2.5. Sample Size Estimation

For the estimation of the sample size, we used the percentage of individuals with occupational exposure to noise as a reference, which was 28% in the control group. Considering that noise exposure is, in no case, a protective factor against vertigo, we estimate a significant difference of 10% of exposed individuals when comparing both groups. With 95% confidence level (1-α) and 90% statistical power, for a unilateral hypothesis test, a total of 377 subjects was deemed the number necessary in each of the two groups.

### 2.6. Sample

A total of 461 patients were initially included in the study sample (220 with MD, 73 with VM, 28 with VN, and 140 with BPPV). The sample comprised 310 women and 151 men. The mean age of patients was 58.6 ± 14.594 years (range: 20.2–91.5 years).

To obtain the control group, we used the national sample of the 6th EWCS-Spain [19], which corresponds to the 2015 edition of the National Survey on Working Conditions and is part of the “Sixth European Working Conditions Survey” carried out by the European Foundation for the Improvement of Living and Working Conditions (Eurofound). In 2015, Eurofound carried out the sixth survey in the series (in operation since 1991). The sixth EWCS was developed in 35 countries. In Spain, this survey interviewed 3364 workers (1714 men and 1650 women) [19]. The mean age of the participants was 43.46 ± 38.513 years. The interviewed working people were randomly selected, comprising a cross-section of society. Survey findings provide information on a broad range of issues, including exposure to physical risks. In the 6th EWCS-Spain, occupations were coded according to ISCO-08 and its Spanish version (NCO-11). This national survey shows a wide-ranging picture of the Spanish economically active population, including the distribution of workers by occupational category. For this study, as a control group, we selected from this sample a group of 400 individuals who were comparable in sex and age with the patients with vertigo.

### 2.7. Statistical Analysis

All data were collected and entered into a database created especially for the purpose. Statistical analysis of results was performed with SPSS version 15.0 for Windows software program. To evaluate if continuous variables showed a normal distribution, the Kolmogorov–Smirnov test was applied. When normality could be assumed, relationships between those variables and the discrete variables were checked by the Student’s *t*-test or ANOVA; a post hoc analysis was performed with Bonferroni-adjusted corrections for multiple comparisons. When continuous variables did not show a normal distribution, the Mann–Whitney non-parametric test was applied to analyze these relationships. The Chi-square test was applied to analyze relationships between discrete variables, and Fisher’s exact test was used to analyze 2 × 2 tables, with the calculation of the odds ratio and 95% confidence intervals. The level of statistical significance for all tests was set at *p* < 0.05.

## 3. Results

The control group included 400 participants. In total, 461 patients affected by vertigo were initially recruited in the current study. We excluded 68 patients corresponding to the category of “housewives, students, and unemployed people” because this category had not been included in the control group (economically active population). Therefore, 393 patients with vertigo were compared to 400 subjects in the control group. Data on the sex and age of the 393 participants distributed into the four diagnostic groups studied are shown in Table 2.

There were no statistically significant differences between both groups in relation to age (Mann–Whitney test, *p* = 0.061). The mean age was 57.46 ± 14.181 years in the group of patients and 56.018 ± 4.484 in the control group. Both groups were also comparable regarding gender (Fisher’s exact test, *p* = 0.461): 244 females vs. 149 males in the group of patients (ratio 1.6/1) and 246 females vs. 154 males in the control group (ratio 1.6/1). Data can be observed in Table 3.

**Figure 1:**
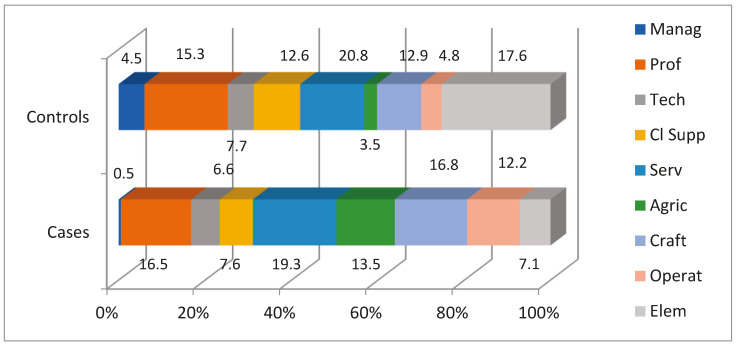
Differences in the distribution of occupations in the case and the control groups (percentage). Manag: managers. Prof: professionals. Tech: technicians and associate professionals. Cl Supp: clerical support workers. Serv: services and sales workers. Agric: skilled agricultural, forestry, and fishery workers. Craft: craft and related trades workers. Operat: plant and machine operators and assemblers. Elem: elementary occupations.

Regarding the occupation of participants, statistically significant differences were detected between the group of patients and the control group (Chi-square, *p* = 4.065 × e^−20^). As can be seen in Figure 1, occupations included in the “managers”, “clerical support workers”, and “elementary occupations” categories showed a lower percentage among patients with vertigo than the general population. By contrast, occupations of “skilled agricultural, forestry and fishery workers”, “craft and related trades workers”, and “plant and machine operators and assemblers” categories showed a higher percentage among patients with vertigo (cases) than the general population (control group).

When occupational exposure to noise was analyzed, statistically significant differences were also observed between both (cases and control) groups (Fisher’s exact test, *p* = 2.97 × e^−10^; OR = 2.595, CI95% (1.916;3.515)). Patients with vertigo were much more exposed to noise (177 patients, 45% of patients were exposed) than participants in the control group (96 subjects, 24% of participants were exposed). When exposure by sex was analyzed in the group of patients, it could be observed that females experienced smaller occupational exposure to noise than males (Fisher’s exact test, *p* = 1.60 × e^−7^; OR = 0.331, CI95% (0.217;0.505)). An important percentage of men affected by vertigo in our sample was occupationally exposed to noise (61.7%, a total of 92 men), while the women with vertigo exposed to noise accounted for 34.8% (a total of 85 women). Based on this fact, exposure to noise in men and women was studied separately, comparing cases vs. controls for each sex. Males suffering from vertigo had higher noise exposures (92 from 149, 61.7%) than control males (50 from 154, 32.5%); these differences were statistically significant (Fisher’s exact test, *p* = 2.53 × e^−7^; OR = 3.357, CI95% (2.094;5.383)). Similar findings were observed among women: 34.8% (85 from 244) of women with vertigo were exposed to noise, whereas only 18.7% of control women were exposed (46 from 246). Differences between both groups of women were also statistically significant (Fisher’s exact test, *p* = 3.85 × e^−5^; OR = 2.324, CI95% (1.535;3.519)).

Statistically significant differences were also found when occupational exposure to vibrations was studied (Fisher’s exact test, *p* = 6.23 × e^−10^; OR = 2.722, CI95% (1.963;3.775)); this exposure was more frequent among patients with vertigo (147 patients, 37.4% of patients were exposed) than in control group (72 subjects, 18% of subjects were exposed). Again, gender differences in occupational vibration exposure were found in the group of patients (Fisher’s exact test, *p* = 0.0003; OR = 0.475, CI95% (0.312;0.723)). Males were also more exposed to this risk (72 men, 48.3% of all men included in our sample) than females (75 women, 30.7% of all women included in our sample). Therefore, differences between cases and control were analyzed by gender. In men, 72 out of 149 males suffering from vertigo were exposed to vibrations (48.3%), whereas 46 of 154 (29.9%) of the control participants were exposed to vibrations. This difference was statistically significant (Fisher’s exact test, *p* = 0.001; OR = 2.195, CI95% (1.370;3.518)). In women, the proportion of those exposed to vibration was higher in women with vertigo (30.7%, 75/244) than in control women (10.6%, 26/246). Again, these observed differences were statistically significant (Fisher’s exact test, *p* = 1.98 × e^−8^; OR = 3.755, CI95% (2.303;6.124)).

**Figure 2:**
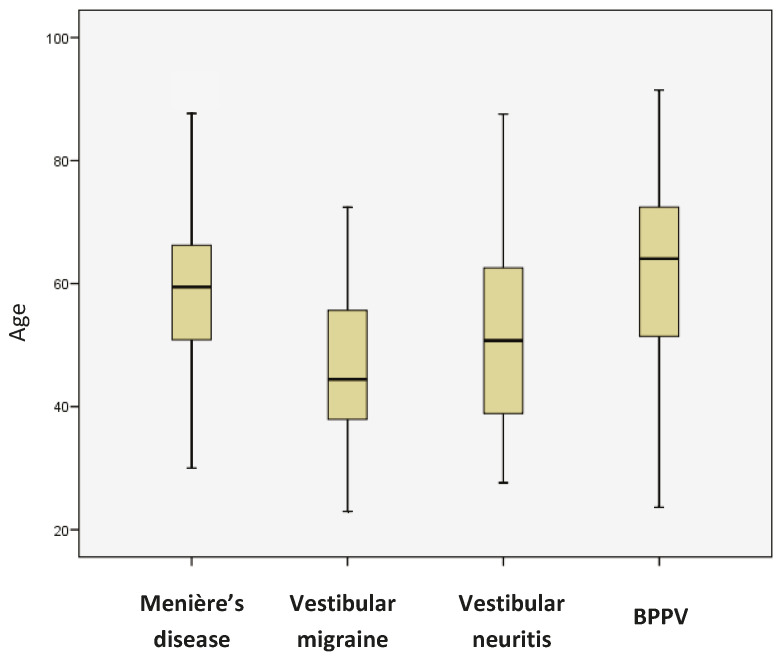
Age distribution of the four diagnostic subgroups.

Since the group of patients with vertigo is heterogeneous in terms of diagnosis, the different diagnostic subgroups were compared in order to determine if exposure to noise and/or vibration was different between them. Figure 2 shows the age distribution of the four diagnostic subgroups. When comparing age (ANOVA, with post hoc Bonferroni-adjusted corrections for multiple comparisons), significant differences were only observed between the subgroups “Menière’s disease” and “vestibular migraine” (*p* = 2.901 × e^−10^), “vestibular migraine” and “BPPV” (*p* = 6.335 × e^−13^), and “vestibular neuritis” and “BPPV” (*p* = 0.009).

**Figure 3:**
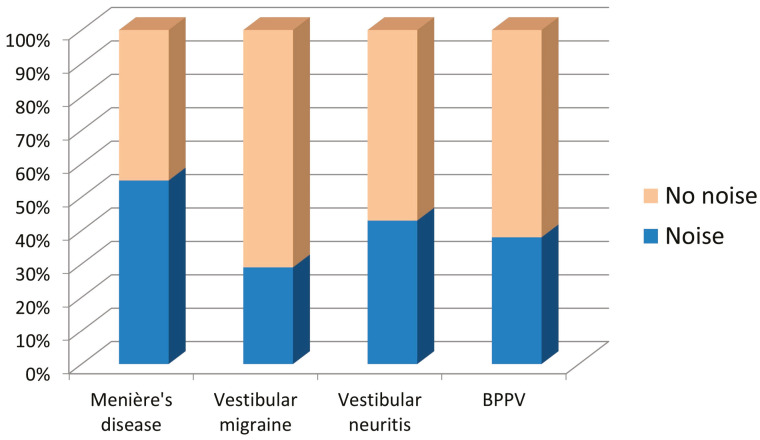
Occupational exposure to noise in the group of patients with vertigo, according to the diagnostic subgroup.

The results obtained regarding exposure to noise can be seen in Table 4. Differences were statistically significant (Chi-square, *p* = 0.001). The percentage of individuals occupationally exposed to noise in the “Menière’s disease” subgroup is higher than in other diagnostic subgroups. The subgroup with the lowest percentage of noise exposure was “vestibular migraine”. These differences can also be seen in Figure 3.

**Figure 4:**
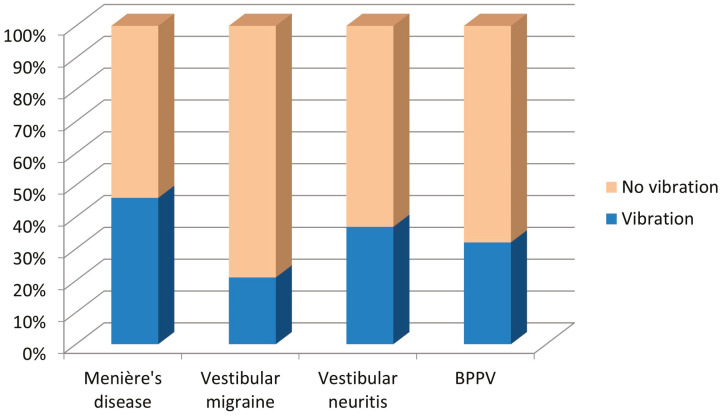
Occupational exposure to vibrations in the group of patients with vertigo, according to the diagnostic subgroup.

Similar results were obtained when occupational exposure to vibrations was studied and compared between diagnostic subgroups (Table 5). Differences were statistically significant (Chi-square, *p* = 0.002). As observed in occupational exposure to noise, patients with Meniere’s disease showed greater occupational exposure to vibrations than the other diagnostic subgroups, while patients with vestibular migraine showed the lowest percentage of exposure to vibrations. These differences can also be observed in Figure 4.

## 4. Discussion

Vertigo and balance disorders are relatively common in the general population and affect both the quality of life and the development of the occupational activities of patients. Several studies reported that the lifestyle of patients and related factors may influence the clinical features of vertigo. The aim of our study was to advance the knowledge of these factors and their association with balance disorders. The relationship between occupation and balance disorders has been scarcely studied, and to date, few studies have been published analyzing only specific occupations [9]. Occupational noise-induced hearing loss is a recognized work-related illness, according to the Spanish List of Occupational Diseases. Recently, it has been proposed that exposure to noise might affect the vestibular system. Regarding mechanical vibrations, the association between this physical risk factor and vestibular symptoms remains unknown. The purpose of this study was to analyze the putative relationship between occupation and related factors, as well as vertigo.

Our study sample initially comprised a total of 461 patients with vertigo, 310 women, and 151 men (female/male ratio = 2.05/1). The mean age of patients was 58.6 years old. Excluded from the study sample, the “housewives, students and unemployed people” group (formed by 97% women), the female/male ratio still showed a female predominance among patients with vertigo (1.6/1). The female predominance observed in our study is in accordance with the findings previously reported in several balance disorders. In Menière’s disease, almost all publications reported a higher prevalence of females, with percentages ranging from 53.2% [20] to 80% [21]. In vestibular migraine, similar findings were reported; it was observed that 84% of patients with vestibular migraine were women [22], according to our results (75% of our patients were women). In BPPV, this higher prevalence of women was also reported (two-fold higher than that of men) [23].

Statistically significant differences were observed when analyzing exposure to noise between men (61.7% exposed) and women (34.8% exposed). Consistent with the results of other studies [24], in our sample, men experienced greater exposure to noise at work than women. Similar results were obtained when studying occupational exposure to vibrations; men were more exposed to vibrations (48.3%) than women (30.7%). Both physical risk factors are related to the occupational categories of patients. Therefore, these differences in exposure to noise and vibrations are likely due to differences in the distribution of occupational categories among males and females. These findings are in accordance with the results of other studies. Worldwide, occupational risks are not distributed evenly among all workers [24]. Males remain represented at higher rates than females in certain occupations with high noise or vibration exposures. According to the 6th EWCS findings, differences in terms of gender and occupation are still significant in Europe. Our study also indicates that differences regarding “work at heights” are even greater between men and women, being exceptional among women. As a consequence of this finding, not only differences between all cases and controls but also differences by gender (cases vs. controls in females and cases vs. controls in males) were analyzed.

When demographic characteristics and occupations of patients with vertigo (excluding the group “housewives, students, and unemployed people” in order to be comparable) were compared to the general population, in addition to the aforementioned differences by gender, there were also observed statistically significant differences in the occupation of the subjects. We consider these findings relevant since they were obtained once the “housewives, students, and unemployed people” group was excluded, taking into account that this group represents a high proportion of patients with vertigo (14.5%) and is mostly comprised of women. “Clerical support workers” and “elementary occupations” are occupational groups represented at lower rates among patients with vertigo, compared to the general population; by contrast, “skilled agricultural, forestry and fishery workers”, “craft and related trades workers” and “plant and machine operators and assemblers” are occupational groups more represented in patients with vertigo than in general population. These results seem to indicate that overall, the prevalence of vertigo may be higher in occupations involving greater physical efforts and handling machinery, whereas more sedentary occupations may be considered as “protective” from suffering vertigo.

Patients with vertigo were more exposed to noise (45%) than control subjects (24%), which seems to indicate that noise might induce or modulate vestibular pathology. Occupational exposure to vibrations was also more prevalent among patients with vertigo (37.4%) compared to the general population (18%). These differences were also detected in the analysis carried out separately by sex. This finding may be likely due to those occupations involving noise exposure also requiring the use of tools and machinery that generate vibrations, and even and likely more importantly, may be due to tools and machinery generating vibrations also producing noise. The influence of occupational exposure to noise and vibrations on vestibular damage remains insufficiently studied. There are papers that analyze the damage produced in the auditory system but without reference to possible vestibular injuries [25]. In our opinion, the relationship that we found between vibrations and vertigo is especially relevant. Although occupational exposure to vibrations has been reported to cause dizziness and motion sickness [12], to our knowledge, there are few manuscripts that suggest the existence of a relationship between vibrations in the workplace and some vestibular diseases [13].

The analysis of occupational exposure to noise and vibrations in the different diagnostic subgroups has been interesting. Menière’s disease is the diagnostic subgroup most associated with these two risk factors. This fact is not surprising since it is known that exposure to loud noise in a patient with Menière’s disease can trigger a vertigo attack. Physical stimuli that act on the inner ear, such as noise and vibrations, may favor the onset of clinical symptoms in previously asymptomatic patients.

Although in a smaller percentage, there is also a relationship between exposure to noise and vibrations and the diagnosis of benign paroxysmal positional vertigo. These occupational risk factors may facilitate, firstly, the detachment of otoconia from the utricle and, secondly, their introduction into the semicircular canals.

This study had three main limitations. Firstly, occupations with higher risk or that require more physical activity could be somewhat oversized in the group of patients with vertigo. These people may be more diligent in seeking medical attention than those with more sedentary work. Secondly, the division between exposed and unexposed (to noise and/or vibrations) is perhaps somewhat simple. Exposure dosing and characteristics (noise level, vibration frequency, acceleration, anatomical region or surface exposed…) would be needed to properly document if exposure risk is elevated. But, in our opinion, our study allows a first approximation to establish a possible etiological relationship between vertigo and exposure to noise and vibrations, which is the essential objective of this study. Once this association has been established, it will be necessary to specify in more detail, in future studies, the exposure thresholds (in intensity, in time, in body surface…) that may favor the development of vertigo and vestibular dysfunction. Thirdly, this study has a monocentric study design. It is possible that the occupational characteristics of the analyzed population (with an economy based essentially on the agricultural, fishing, and tourist services sectors) are different from those in other areas in our country.

## 5. Conclusions

A relevant difference in occupational group distribution was observed in patients with vertigo compared to the general population. There was an association between vertigo and certain occupations involving physical efforts and higher exposure to certain risk factors such as noise and vibrations. We consider that collective and individual preventive and protective measures must be improved to safeguard the health of workers and ensure a high degree of protection against noise or vibrations (e.g., reducing the time of exposure). We propose that these protective and preventive measures might reduce the incidence or improve the evolution of certain diseases that cause vertigo.

## Figures and Tables

**Table 1:** Major occupational groups according to the International Standard Classification of Occupations, 2008 (ISCO-08) and its Spanish version (National Classification of Occupations, NCO-11) [16,17].

Group	Description
1	Managers
2	Professionals
3	Technicians and associate professionals
4	Clerical support workers
5	Service and sales workers
6	Skilled agricultural, forestry, and fishery workers
7	Craft and related trades workers
8	Plant and machine operators and assemblers
9	Elementary occupations
0	Armed forces occupations

**Table 2:** Patients distributed by sex and age in diagnostic groups.

Diagnostic Group	Women	Men	Total	Mean Age (±SD)
Menière’s disease	105	88	193	59.0 (±11.894)
Vestibular migraine	47	16	63	46.1 (±12.068)
Vestibular neuritis	14	7	21	52.0 (±15.655)
Benign paroxysmal positional vertigo	78	38	116	62.0 (±15.081)
Total	244	149	393	57.5 (±14.181)

**Table 3:** Distribution by sex in case and control groups.

	Women n (%)	Men n (%)	Total
Cases	244 (62.1)	149 (37.9)	393
Controls	246 (61.5)	154 (38.5)	400
Total	490 (61.8)	303 (38.2)	793

**Table 4:** Occupational exposure to noise in the different diagnostic subgroups (percentage).

Diagnostic Group	Noise n (%)	No Noise n (%)	Total
Menière’s disease	106 (54.9)	87 (45.1)	193
Vestibular migraine	18 (28.6)	45 (71.4)	63
Vestibular neuritis	9 (42.9)	12 (57.1)	21
Benign paroxysmal positional vertigo	44 (37.9)	72 (62.1)	116
Total	177 (45)	216 (55)	393

**Table 5:** Occupational exposure to vibrations in the different diagnostic subgroups (percentage).

Diagnostic Group	Vibration n (%)	No Vibration n (%)	Total
Menière’s disease	89 (46.1)	104 (53.9)	193
Vestibular migraine	13 (20.6)	50 (79.4)	63
Vestibular neuritis	8 (38.1)	13 (61.9)	21
Benign paroxysmal positional vertigo	37 (31.9)	79 (68,1)	116
Total	147 (37.4)	246 (62.6)	393

## Data Availability

The data presented in this study are available on request from the corresponding author due to privacy, legal and ethical reasons.
